# Knowledge, attitude, and practice of patients with oral diseases toward oral examinations: a cross-sectional survey study

**DOI:** 10.3389/fpubh.2024.1424503

**Published:** 2024-07-02

**Authors:** Wei Wang

**Affiliations:** ^1^Shanghai Engineering Research Center of Tooth Restoration and Regeneration and Tongji Research Institute of Stomatology, Shanghai, China; ^2^Department of Clinical Medical Laboratory, Tongji University Stomatological Hospital, Shanghai, China; ^3^Dental School, Tongji University, Shanghai, China

**Keywords:** knowledge, attitude, practice, oral diseases, patients, cross-sectional study

## Abstract

**Background:**

Properly adhering to oral hygiene and medical care is an important public health issue. Several studies examined the knowledge, attitudes, and practices (KAP) toward oral care in various populations and generally reported relatively sufficient knowledge but unfavorable attitudes and poor practice. However, no previous studies have examined the KAP toward oral examinations among Chinese patients with oral diseases. This study aimed to examine the KAP toward oral examinations among patients with oral diseases in China.

**Methods:**

This cross-sectional study was conducted in patients with oral diseases who visited The Affiliated Stomatological Hospital of Tongji University between December 2023 and February 2024. Data collection and KAP scores assessment were performed using a self-designed questionnaire.

**Results:**

A total of 519 valid questionnaires were included, with 292 females. The mean knowledge, attitude, and practice scores were 6.42 ± 2.47 (possible range: 0–9 points), 35.04 ± 5.68 (possible range: 10–50 points), and 16.22 ± 2.05 (possible range: 4–20 points), respectively, indicating sufficient knowledge, positive attitudes, and proactive practice. Pearson’s correlation analysis showed that knowledge was positively correlated to attitude (*r* = 0.468, *p* < 0.001) and practice (OR = 0.416, *p* < 0.001). Attitude was positively correlated to the practice (*r* = 0.503, *p* < 0.001). Moreover, the structural equation model showed that knowledge influenced attitude (estimate = 1.010, *p* < 0.001) and practice (estimate = 0.169, *p* < 0.001). Attitude influenced practice (estimate = 0.122, *p* < 0.001). The frequency of oral examination per year influenced knowledge (estimate = −0.761, *p* < 0.001) and practice (estimate = −0.515, *p* < 0.001). Expenses for oral disease per year influenced attitude (estimate = 0.537, *p* < 0.001).

**Conclusion:**

Patients with oral disease might have sufficient knowledge, positive attitude, and proactive practice toward oral examinations. Specific knowledge items were identified to require improvements.

## Introduction

Oral diseases encompass a wide variety of diseases and conditions, including odontogenic diseases (e.g., dental caries and pulpitis) (1, 2), tooth fracture (3), crack, temporomandibular joint disease (4), tooth implantation (5), oral mucosal diseases (e.g., oral ulcers) (6), periodontal diseases (e.g., gingivitis) (7), maxillofacial deformities, and tumors (8, 9). Despite their vast differences in etiology, risk factors, incidence, prevalence, and management, most of these conditions have in common that they require an oral examination for diagnosis, examinations during management until resolution, and regular follow-up examinations to check for recurrence and allow early treatment. Gingivitis can be diagnosed by oral hygienists and can be reversed by adequate oral hygiene, but the patients might need guidance regarding oral hygiene. Maintaining good oral health is important since periodontal diseases are risk factors for non-communicable diseases like cardiovascular diseases, diabetes, cancers, and adverse pregnancy outcomes, among others (10–12).

The adequate management of these conditions first requires the patient to realize that something is wrong and seek consultation. After that, a proper understanding of the importance of adherence to oral examinations during the management and follow-up of the condition is also required. Indeed, several oral conditions have a high risk of recurrence after successful management. Repeated recurrences, especially if the recurrence is not managed early, are associated with significant morbidity and treatment costs and can increase the risk of mortality (e.g., cancer, of course, but also the risk of endocarditis in patients with untreated dental abscess) (13–15).

Hence, properly adhering to oral hygiene and medical care is an important public health issue (16). Knowledge, attitude, and practice (KAP) studies are designed to provide quantitative and qualitative information about the gaps, misunderstandings, and misconceptions that represent barriers to adequately implementing a specific subject in a specific population (17, 18). Such studies are particularly useful in identifying specific points that should be addressed in educational interventions. Several studies examined the KAP toward oral care in various populations and generally reported relatively sufficient knowledge but unfavorable attitudes and poor practice (19–24). Still, no previous studies have examined the KAP in relation to oral examinations among Chinese patients with oral diseases. Therefore, this study aimed to explore the KAP toward oral examinations among patients with oral diseases.

## Materials and methods

### Study design and setting

This cross-sectional study was conducted in patients with oral diseases at The Affiliated Stomatological Hospital of Tongji University between December 2023 and February 2024.

### Participants

The inclusion criterion was patients with oral diseases who visited the hospital. The exclusion criteria were (1) medical professionals, (2) <18 years of age, (3) questionnaires with contradictory answers, or (4) questionnaires with all answers selected with the same option. This study was approved by the Ethics Committee of Tongji University Affiliated Stomatological Hospital ([2023]-SR-36). Written informed consent was obtained by all participants before completing the questionnaire. For the electronic survey, the informed consent statement was the first step of the questionnaire; electronic consent was necessary to access the questionnaire itself.

### Variables

The basic information section included gender, age, residence, education, employment, income, marital status, smoking, oral health expenses, medical insurance, course of oral diseases, types of oral disease, tooth brushing frequency, tooth cleaning frequency, oral examination frequency, and history of head and neck electrotherapy.

### Data sources and measurements

The questionnaire was designed based on “*Prevention, Diagnosis, and Treatment of Oral Diseases*.” The questionnaire was reviewed by experts in the field to clarify the questions (content value). A small-scale pilot study (44 participants) showed a Cronbach’s *α* of 0.810, indicating good internal consistency. The participants of the pilot study were invited to indicate any unclear questions (face value).

The final questionnaire was in Chinese and included information collected from four dimensions, comprising 41 items. The basic information section included 16 items. The knowledge dimension included nine items, with 1 point awarded for a correct answer and 0 points for an incorrect or unclear answer (possible range: 0–9 points). A score <5 indicated poor knowledge, and 5–9 indicated sufficient knowledge. The attitude dimension included 10 items, with options ranging from negative to positive and scored from 1 to 5 (possible range: 10–50 points), with a score of 10–20 indicating a negative attitude, 21–35 indicating a neutral attitude, and >35 indicating a positive attitude. The practice dimension included five items. Items P1–P4 were scored from negative (1 point) to positive (5 points) (possible range: 4–20 points), with a score of 4–8 indicating poor practice, 9–15 indicating average practice, and 16–20 indicating good practice. Item P5 was a multiple-choice question and was not scored but was described as a separate categorical variable.

Two research assistants, both professional interns in the field of medical laboratory, were trained to handle recruitment and the questionnaire. The research assistants contacted the patients through the patient contact information disclosed in the hospital’s online platform or communicated with the patients in person in the office and recruited and issued questionnaires to the eligible patients. The questionnaires included paper questionnaires, electronic questionnaires, and questionnaires pushed by the hospital platform.

### Bias

In order to ensure that the participants could correctly understand the questionnaire content, the writing was in the form of clear and concise questions, providing definitions and examples. In addition, the research assistants could provide supplementary explanations or answer participants’ questions during the questionnaire delivery process.

Incomplete questionnaires and those with all KAP items answered using the same option (e.g., all first options) were considered invalid. For offline questionnaires, the participants could not go home with the questionnaire and bring it back later; doing so led to questionnaire exclusion. For online questionnaires, response time <60 s or >1,800 s led to questionnaire exclusion. Only one questionnaire could be submitted from a given IP address.

### Study size

The sample size was estimated based on Ni’s method for quantitative surveys, i.e., 5–10 times the number of KAP items in the survey (25). The survey included 23 items. Therefore, the minimal sample size was 115–230.

### Quantitative variables

Descriptive analysis was used to describe the demographic data of the study participants and their KAP scores, using means ± standard deviations for continuous variables and *n* (%) for categorical data. Since the sample size of this paper was relatively large, based on the central limit theorem, it was assumed that the data in this study were normally distributed.

### Statistical analysis

SPSS 22.0 (IBM Corp., Armonk, NY, USA) was used for statistical analysis. In order to compare the differences in KAP scores among study participants with different demographic characteristics, the t-test (two-group comparisons) or ANOVA (comparisons of more than two groups) were used for continuous variables that met the normality assumption, while the Wilcoxon–Mann–Whitney test (comparison of two groups) or the Kruskal–Wallis analysis of variance (multiple groups) were used. Pearson’s correlation analysis was performed to examine the correlations among KAP dimensions. Structural equation modeling (SEM) was used to explore the path relationships between KAP and basic information. The SEM was based on the hypotheses that (1) knowledge influences attitude, (2) knowledge influences practice, (3) attitude influences practice, (4) the frequency of oral examinations per year influences knowledge, attitude, and practice, (5) the course of oral diseases influences knowledge, attitude, and practice, and (6) and the expenses for oral diseases per year influences knowledge, attitude, and practice. Two-sided *p*-values <0.05 were considered statistically significant.

## Results

**Figure 1:**
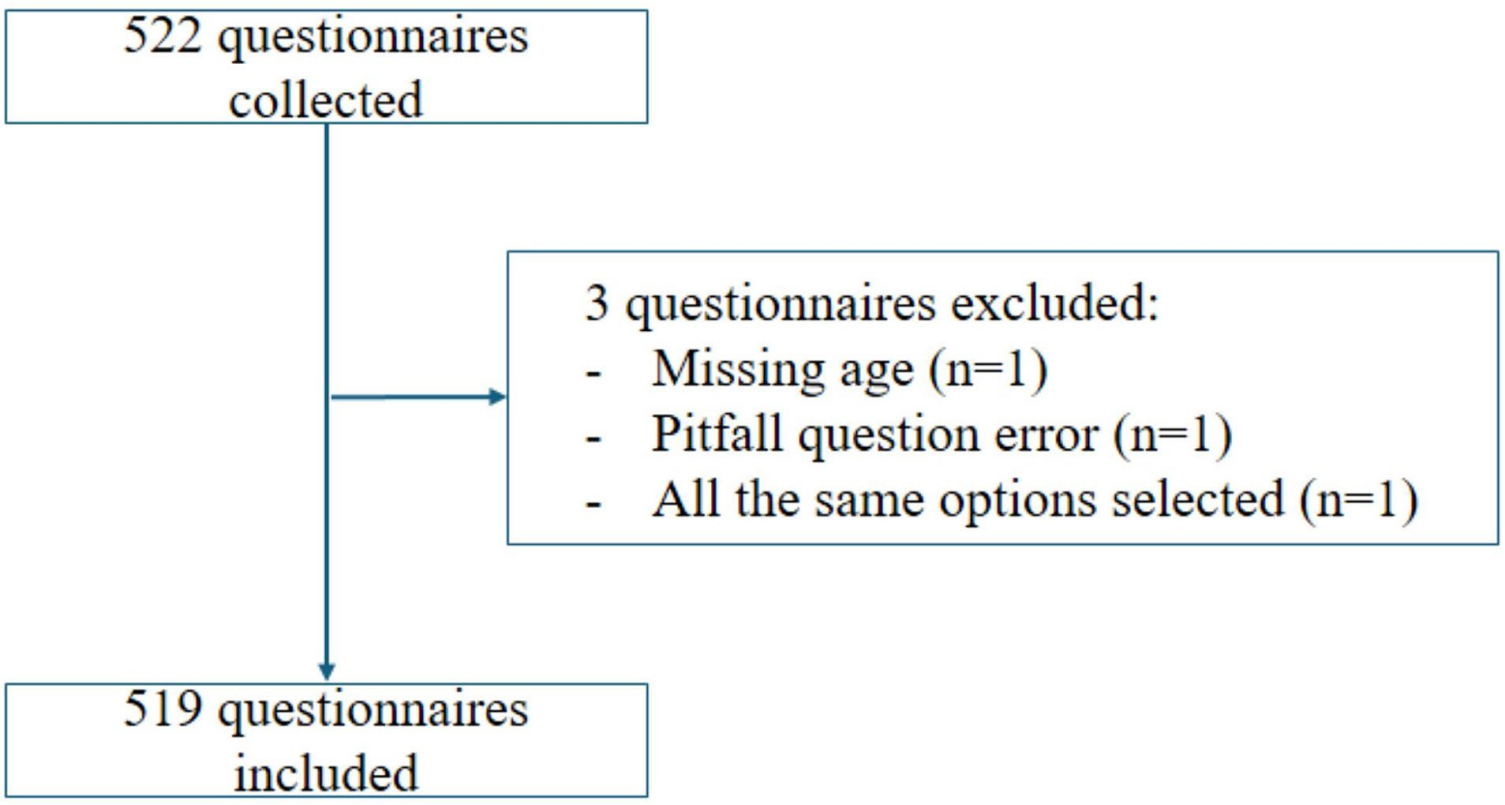
Participant flowchart.

**Figure 2:**
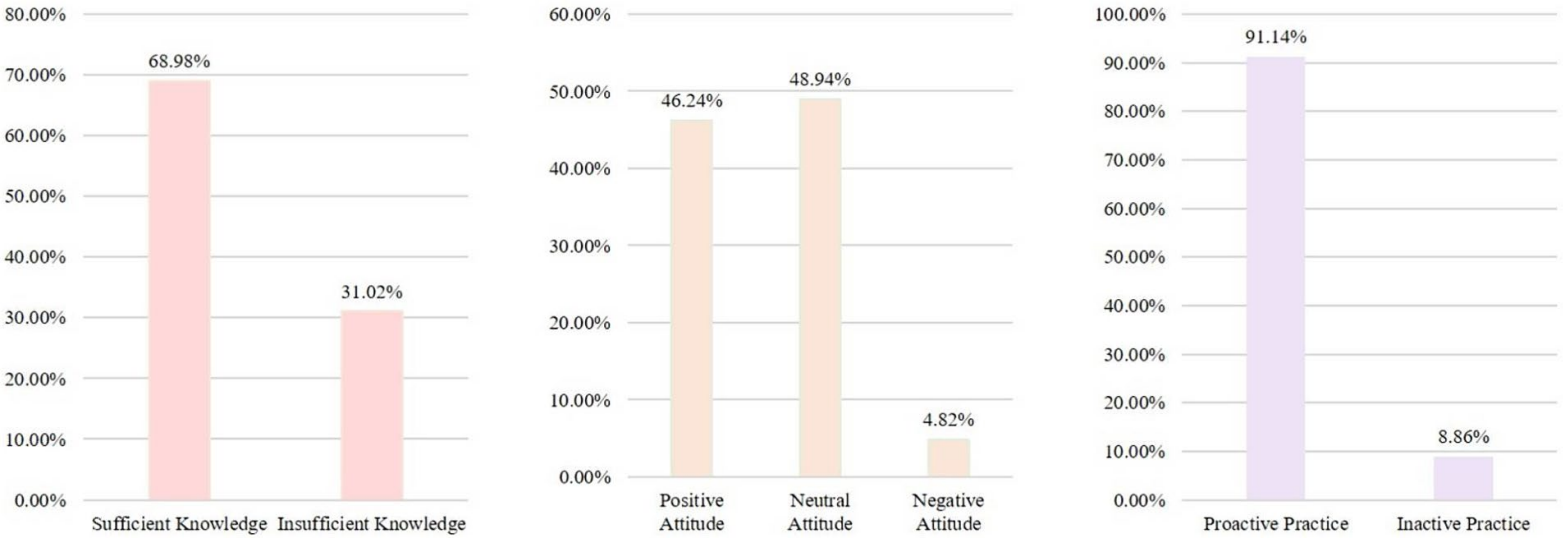
Histogram of the score distribution. K: There are nine questions, with scores ranging from 0 to 5 indicating insufficient knowledge and scores ranging from 6 to 9 indicating sufficient knowledge. A: There are 10 questions, with scores ranging from 10 to 26 indicating a negative attitude, scores ranging from 27 to 36 indicating a neutral attitude, and scores ranging from 37 to 50 indicating a positive attitude. P: There are four questions, with scores ranging from 4 to 13 indicating inactive practice and scores ranging from 14 to 20 indicating proactive practice.

This study collected 522 questionnaires, but one was excluded for missing age information, one for contradictory answers in the pitfall question, and one for the same answer being selected for all KAP questions. Finally, 519 valid questionnaires were included for analysis (Figure 1). There were 56.26% of female patients, with 18–30 years of age (35.26%). The mean knowledge, attitude, and practice scores were 6.42 ± 2.47 (possible range: 0–9 points), 35.04 ± 5.68 (possible range: 10–50 points), and 16.22 ± 2.05 (possible range: 4–20 points), respectively, indicating sufficient knowledge, positive attitudes, and good practice (Table 1). Among them, 68.98% had sufficient knowledge, 46.24% had a positive attitude, and 91.14% had a proactive practice (Figure 2).

**Table 1:** Characteristics of the participants.

Variables	*n* (%)	Knowledge score		Attitude score		Practice score	
		Mean ± SD	*p*	Mean ± SD	*p*	Mean ± SD	*p*
Total	519	6.42 ± 2.47		35.04 ± 5.68		16.22 ± 2.05	
Gender			0.714		0.954		0.584
Male	227 (43.74)	6.38 ± 2.59		35.02 ± 5.76		16.28 ± 1.89	
Female	292 (56.26)	6.46 ± 2.37		35.05 ± 5.62		16.18 ± 2.16	
Age, years			0.148		0.289		0.263
18–30	183 (35.26)	6.46 ± 2.31		35.48 ± 5.65		16.19 ± 1.88	
31–35	175 (33.72)	6.65 ± 2.39		35.07 ± 5.87		16.41 ± 2.10	
≥36	161 (31.02)	6.13 ± 2.70		34.51 ± 5.49		16.05 ± 2.17	
Residence			0.062		0.206		0.316
Non-urban	66 (12.72)	6.50 ± 2.46		35.16 ± 5.62		16.26 ± 2.07	
Urban	453 (87.28)	5.89 ± 2.48		34.21 ± 6.01		15.98 ± 1.87	
Education			0.015		0.838		0.402
High school/vocational school and below	36 (6.94)	5.53 ± 2.47		35.06 ± 6.85		15.92 ± 2.23	
College/undergraduate	435 (83.82)	6.42 ± 2.49		34.99 ± 5.68		16.21 ± 2.05	
Graduate and above	48 (9.25)	7.10 ± 2.01		35.50 ± 4.75		16.52 ± 1.91	
Employment			0.897		0.603		0.769
Employed	481 (92.68)	6.42 ± 2.48		35.00 ± 5.72		16.21 ± 2.08	
Unemployment or others	38 (7.32)	6.47 ± 2.26		35.50 ± 5.12		16.32 ± 1.60	
Per capita monthly income, Yuan			0.049		0.143		0.056
<5,000	55 (10.60)	5.67 ± 2.29		34.60 ± 6.59		15.89 ± 2.12	
5,000–10,000	192 (36.99)	6.32 ± 2.43		34.56 ± 5.56		16.08 ± 2.13	
10,000–20,000	170 (32.76)	6.62 ± 2.48		35.06 ± 5.79		16.21 ± 1.85	
>20,000	102 (19.65)	6.71 ± 2.54		36.13 ± 5.10		16.69 ± 2.14	
Marital status			0.010		0.573		0.109
Single	136 (26.20)	5.90 ± 2.61		34.64 ± 5.55		15.90 ± 2.01	
Married	381 (73.41)	6.62 ± 2.39		35.17 ± 5.74		16.33 ± 2.05	
Divorced	2 (0.39)	5.00 ± 0.00		37.00 ± 2.83		16.50 ± 3.54	
Smoking			0.242		0.005		0.005
Never smoked	359 (69.17)	6.48 ± 2.43		34.97 ± 5.56		16.14 ± 2.12	
Former smoker	68 (13.10)	5.96 ± 2.59		33.51 ± 5.41		15.87 ± 1.87	
Current smoker	92 (17.73)	6.53 ± 2.50		36.45 ± 6.06		16.82 ± 1.80	
Expenses for oral disease per year, Yuan			<0.001		<0.001		<0.001
Less than 100	71 (13.68)	5.30 ± 3.17		31.01 ± 5.36		14.89 ± 1.91	
100–500	108 (20.81)	5.82 ± 2.42		33.98 ± 4.47		15.95 ± 2.02	
500–1,000	106 (20.42)	6.46 ± 2.40		35.39 ± 5.61		16.16 ± 1.96	
1,000–5,000	148 (28.52)	7.40 ± 1.85		37.58 ± 5.46		17.09 ± 1.80	
More than 5,000	72 (13.87)	6.74 ± 2.01		35.96 ± 5.17		16.60 ± 1.87	
Not sure	14 (2.70)	4.57 ± 2.85		29.36 ± 4.03		14.36 ± 1.86	
Medical insurance (multiple choice)			-		-		-
Urban employee basic medical insurance	442 (85.16)	6.49 ± 2.49		34.93 ± 5.83		16.17 ± 2.09	
New rural cooperative medical insurance	75 (14.45)	6.37 ± 2.27		34.72 ± 6.31		16.63 ± 1.67	
Urban resident basic medical insurance	117 (22.54)	6.05 ± 2.40		33.94 ± 4.95		16.08 ± 1.93	
Retired cadre medical insurance	10 (1.93)	6.80 ± 2.10		31.60 ± 4.27		16.70 ± 2.45	
Commercial insurance	128 (24.66)	6.94 ± 2.21		36.15 ± 6.24		16.95 ± 2.12	
No insurance	4 (0.77)	4.75 ± 2.22		33.25 ± 2.22		15.00 ± 0.82	
Course of oral diseases			<0.001		<0.001		<0.001
Less than 1 year	146 (28.13)	5.71 ± 2.91		33.04 ± 5.00		15.47 ± 1.93	
1–3 years	195 (37.57)	7.02 ± 1.94		35.87 ± 5.73		16.57 ± 2.10	
3–5 years	83 (15.99)	6.75 ± 2.40		35.96 ± 6.01		16.60 ± 2.04	
More than 5 years	95 (18.30)	6.03 ± 2.44		35.60 ± 5.58		16.34 ± 1.84	
Type of oral diseases			0.361		0.213		0.363
Odontogenic diseases, such as dental caries, pulpitis	156 (30.06)	6.71 ± 2.28		35.67 ± 5.70		16.38 ± 1.77	
Tooth fracture/crack/temporomandibular joint disease/tooth implantation	66 (12.72)	6.15 ± 2.28		34.53 ± 5.72		15.86 ± 2.25	
Oral mucosal diseases, such as oral ulcers	105 (20.23)	6.17 ± 2.46		34.48 ± 5.38		16.08 ± 2.14	
Periodontal disease, such as gingivitis	187 (36.03)	6.44 ± 2.66		35.11 ± 5.81		16.31 ± 2.14	
Other, such as maxillofacial deformities, tumors	5 (0.96)	5.80 ± 2.86		31.20 ± 4.02		15.60 ± 1.82	
Frequency of brushing teeth per day			0.321		<0.001		0.005
Once a day	85 (16.38)	6.27 ± 2.30		32.94 ± 5.33		15.59 ± 2.03	
Twice a day	413 (79.58)	6.41 ± 2.52		35.32 ± 5.59		16.31 ± 2.03	
Three times a day or more	20 (3.85)	7.40 ± 1.85		38.15 ± 6.71		17.15 ± 2.03	
Do not brush teeth	1 (0.19)	6.00		35.00		16.00	
Frequency of teeth cleaned per year			<0.001		<0.001		<0.001
None	165 (31.79)	5.55 ± 2.81		32.71 ± 5.92		15.22 ± 1.99	
1–2 times	331 (63.78)	6.85 ± 2.20		36.05 ± 5.63		16.68 ± 1.95	
More than 2 times	23 (4.43)	6.61 ± 1.80		37.26 ± 3.67		16.83 ± 1.30	
Frequency of oral examination per year			<0.001		<0.001		<0.001
Once a month	20 (3.85)	6.65 ± 1.84		34.45 ± 7.13		17.35 ± 1.50	
Every 6 months	176 (33.91)	7.01 ± 2.13		36.85 ± 5.63		16.95 ± 1.87	
Once a year	239 (46.05)	6.49 ± 2.35		35.12 ± 5.06		16.06 ± 1.93	
Never	84 (16.18)	4.96 ± 2.99		31.15 ± 5.20		14.89 ± 2.0	
Received head and neck electrotherapy			0.120		0.930		0.013
Yes	61 (11.75)	6.89 ± 1.93		35.10 ± 6.54		16.84 ± 1.73	
No	458 (88.25)	6.36 ± 2.53		35.03 ± 5.56		16.14 ± 2.08	

**Figure 3:**
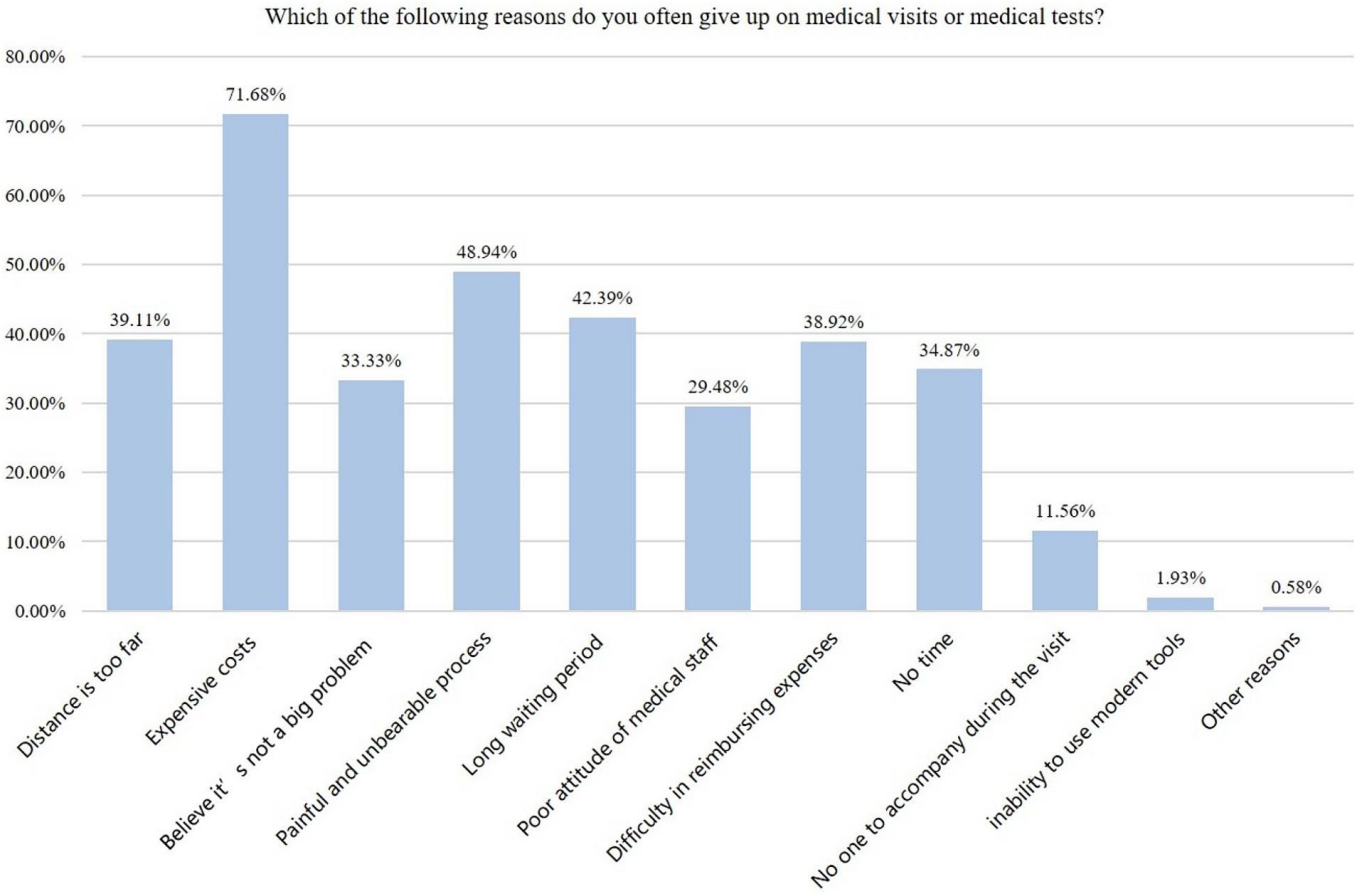
Barriers to medical examinations.

For the knowledge dimension, the highest correct rate was for item K4 (81.50%; “Before root canal treatment, patients need to undergo clinical examinations to determine the size of the carious tissue and whether the dental nerve is vital, such as blood tests and imaging examinations”), while the lowest correct rate was for item K2 (48.36%; “Periodontitis does not require blood tests”) (Table 2). Among the items in the attitude dimension, the item with the highest proportion of positive responses was A1 (“You attach great importance to oral diseases, so you believe that regular oral examinations should be conducted”), while the lowest was observed for A4 (“You believe that the treatment of oral diseases will increase the risk of infectious diseases”) (Table 3). Among the items in the practice dimension, the item with the highest proportion of positive responses was P3 (“You follow the doctor’s advice on whether to undergo blood tests or other medical examinations”), while the lowest was P4 (“Even if there are no obvious oral symptoms, you regularly have your teeth cleaned and undergo blood tests or other medical examinations if necessary”) (Table 4). In addition, the main barrier to practice was the cost (Figure 3).

**Table 2:** Knowledge dimension.

Items	Correct, *n* (%)
K1. Examinations for oral diseases mainly include blood tests, oral microbiological sampling, and imaging examinations.	400 (77.07)
K2. Periodontitis does not require blood tests.	251 (48.36)
K3. Oral surgery, such as tooth extraction, generally requires blood tests, such as routine blood tests, coagulation tests, C-reactive protein tests, and infectious disease tests.	377 (72.64)
K4. Before root canal treatment, patients must undergo clinical examinations to determine the size of the carious tissue and whether the dental nerve is vital, such as blood tests and imaging examinations.	423 (81.50)
K5. Before treating many oral diseases, such as root canal treatment, tooth extraction, dental implantation, and teeth cleaning, it is necessary to determine whether the patient has hypertension, diabetes, heart disease, or has undergone heart valve surgery, etc.	414 (79.77)
K6. Blood tests are generally required before root canal treatment to help doctors understand the possible causes and effects of the disease, which helps establish the correct treatment plan.	422 (81.31)
K7. Tooth implantation is not 100% successful. In order to ensure the success rate of tooth implantation, it is recommended that blood tests be performed before implantation to exclude some systemic diseases that may cause implantation failure.	408 (78.61)
K8. Even if there is periodontal disease before tooth implantation, it does not affect the success rate of implantation.	288 (55.49)
K9. Oral inflammations such as periodontitis, oral mucosal diseases, and peri-implantitis generally require blood tests.	351 (67.63)

For the purpose of this publication, the items were directly translated from Chinese without a formal validation and translation process.

**Table 3:** Attitude dimension.

Items	Strongly agree, *n* (%)	Agree, *n* (%)	Neutral, *n* (%)	Disagree, *n* (%)	Strongly disagree, *n* (%)
A1. You attach great importance to oral diseases, so you believe that regular oral examinations should be conducted.	193 (37.19)	248 (47.78)	69 (13.29)	8 (1.54)	1 (0.19)
A2. You feel worried and anxious about the examination and treatment of tooth extraction and tooth implantation.	75 (37.38)	194 (37.38)	123 (23.70)	107 (20.62)	20 (3.85)
A3. You believe that as long as you brush your teeth frequently, oral diseases can be improved, and frequent checkups are unnecessary.	24 (4.62)	102 (19.65)	124 (23.89)	213 (41.04)	56 (10.79)
A4. You believe that treating oral diseases will increase the risk of infectious diseases.	40 (7.71)	160 (30.83)	148 (28.52)	131 (25.24)	40 (7.71)
A5. You believe that oral disease prevention is more important than treatment, so regular examinations are needed.	156 (30.06)	286 (55.11)	65 (12.52)	11 (2.12)	1 (0.19)
A6. You believe that blood tests are unnecessary for diagnosing oral diseases.	25 (4.82)	87 (16.76)	129 (24.86)	220 (42.39)	58 (11.18)
A7. Due to radiation, you are unwilling to undergo imaging examinations for oral diseases.	22 (4.24)	75 (14.45)	111 (21.39)	233 (44.89)	78 (15.03)
A8. You believe that blood tests are important for treating oral diseases.	100 (19.27)	252 (48.55)	124 (23.89)	39 (7.51)	4 (0.77)
A9. If you do not undergo tooth extraction or implantation, you are unwilling to undergo examinations.	29 (5.59)	125 (24.08)	123 (23.70)	179 (34.49)	63 (12.14)
A10. You are willing to undergo regular oral examinations to prevent oral diseases.	120 (23.12)	259 (49.90)	104 (20.04)	34 (6.55)	2 (0.39)

For the purpose of this publication, the items were directly translated from Chinese without a formal validation and translation process.

**Table 4:** Practice dimension.

Items	Always, *n* (%)	Frequently, *n* (%)	Sometimes, *n* (%)	Occasionally, *n* (%)	Never, *n* (%)
1. You want to understand why blood tests are necessary for the treatment, tooth extraction, or tooth implantation of oral diseases.	154 (29.67)	302 (58.19)	51 (9.83)	10 (1.93)	2 (0.39)
2. You want to understand which oral diseases require blood tests or other medical examinations.	176 (33.91)	275 (52.99)	60 (11.56)	7 (1.35)	1 (0.19)
3. You follow the doctor’s advice on whether to undergo blood tests or other medical examinations.	216 (41.62)	255 (49.13)	38 (7.32)	8 (1.54)	2 (0.39)
4. Even if there are no obvious oral symptoms, you regularly have your teeth cleaned and undergo blood tests or other medical examinations if necessary.	89 (17.15)	211 (40.66)	143 (27.55)	65 (12.52)	11 (2.12)

For the purpose of this publication, the items were directly translated from Chinese without a formal validation and translation process.

Pearson’s correlation analysis showed that knowledge was positively correlated to attitude (*r* = 0.468, *p* < 0.001) and practice (OR = 0.416, *p* < 0.001). The attitude scores were positively correlated to the practice scores (*r* = 0.503, *p* < 0.001) (Table 5).

**Table 5:** Correlation analysis.

	Knowledge	Attitude	Practice
Knowledge	1		
Attitude	0.468 (*p* < 0.001)	1	
Practice	0.416 (*p* < 0.001)	0.503 (*p* < 0.001)	1

**Figure 4:**
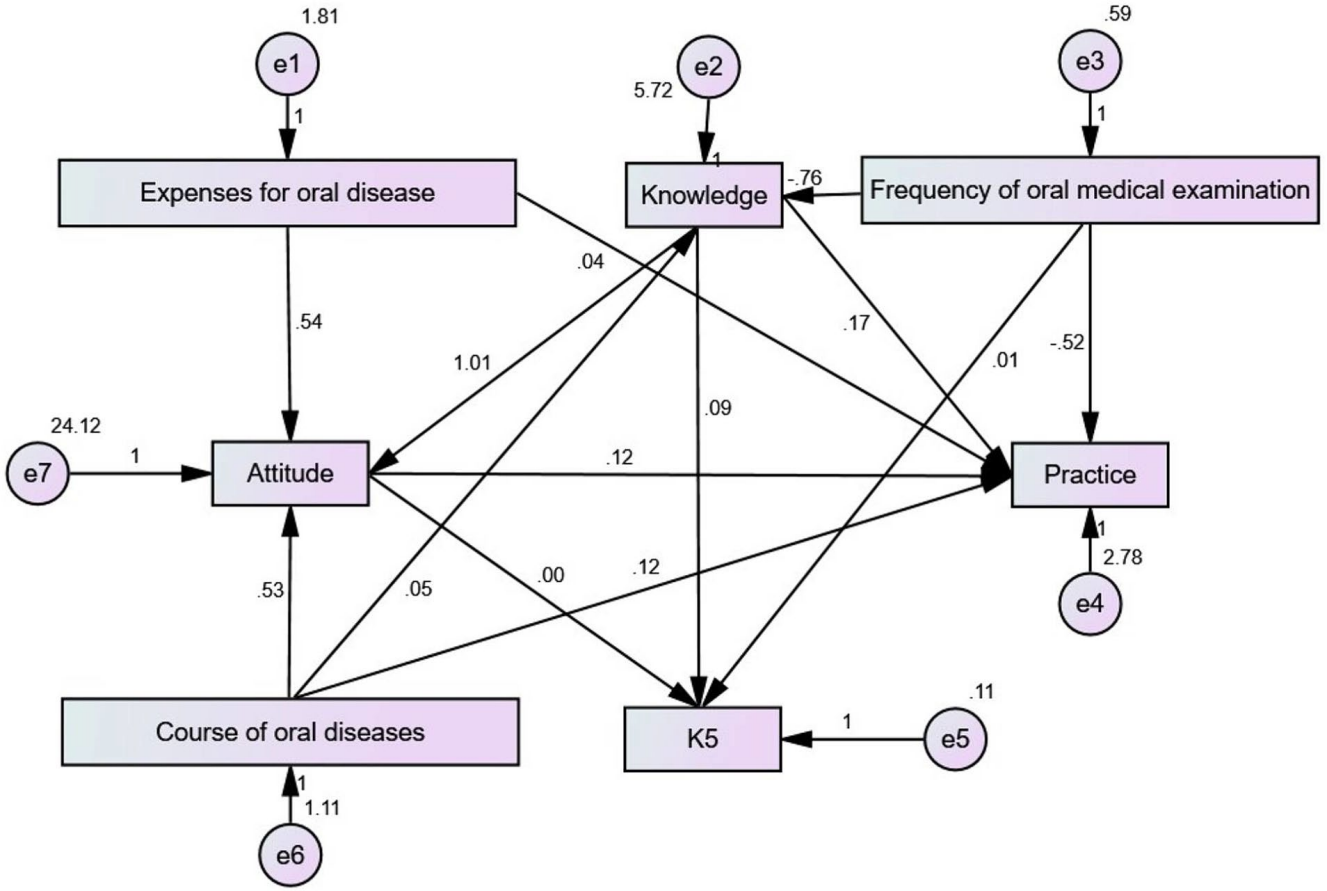
Structural equation model.

SEM showed that knowledge influenced attitudes (estimate = 1.010, *p* < 0.001) and practice (estimate = 0.169, *p* < 0.001). Attitude influenced practice (estimate = 0.122, *p* < 0.001). The frequency of oral examination influenced knowledge (estimate = −0.761, *p* < 0.001) and practice (estimate = −0.515, *p* < 0.001). Expenses for oral disease per year influenced attitude (estimate = 0.537, *p* < 0.001) (Table 6 and Figure 4).

**Table 6:** SEM.

Path relationships			Estimate	*p*
K	←	Frequency of oral examination per year	−0.761	<0.001
K	←	Course of oral diseases	0.049	0.621
A	←	Expenses for oral disease per year	0.537	<0.001
A	←	K	1.010	<0.001
A	←	Course of oral diseases	0.526	0.010
P	←	Frequency of oral examination	−0.515	<0.001
P	←	K	0.169	<0.001
P	←	Expenses for oral disease per year	0.041	0.457
P	←	A	0.122	<0.001
P	←	Course of oral diseases	0.118	0.092
K5	←	K	0.094	<0.001
K5	←	Frequency of oral examination per year	0.012	0.541
K5	←	A	−0.003	0.379

## Discussion

This cross-sectional study examined the KAP toward oral examinations among Chinese patients with oral diseases. Chinese patients with oral disease have sufficient knowledge, positive attitudes, and good practice toward oral diseases. Knowledge influenced the attitudes, practice, and attitude influenced practice. The frequency of oral examinations per year influenced knowledge and practice. Expenses for oral disease per year influenced attitude. The findings could help design interventions to improve oral health in general. This study identified specific knowledge items that could be improved through educational interventions, especially regarding the need for blood tests in some oral diseases and the possible complications of diseases on tooth implantation. The present study showed that improvements in knowledge should also translate into improvements in attitudes and practice.

Oral health is a major public health concern because it significantly impacts healthcare expenses, quality of life, and the risk of complications and other diseases (16, 26). For example, an untreated dental abscess can cause endocarditis, or an apparently benign oral lesion can be a developing oral cancer (13–15). Therefore, oral health requires the individuals’ active participation, with proper hygiene habits (brushing teeth, flossing, and looking for potential lesions), undergoing regular teeth cleaning and dentist consultation, and consulting in the presence of a problem (16, 26). The present study revealed good knowledge, positive attitudes, and proactive practice in patients with oral diseases. It contrasts with several previous studies that reported variable knowledge but generally negative attitudes and poor practice toward oral care (19–24). Of course, adherence to oral health can vary widely among countries based on the socioeconomic status, healthcare literacy of the general population, oral health services available, etc. In addition, these previous studies (19–24) were performed in specific populations (e.g., nurses, patients with heart diseases, dental hygienists, parents, adolescents, and married couples) and conditions (oral health, oral hygiene, and oral cancer), limiting their scope and generalizability.

In China, an earlier study published in 2007 showed that 1,590 Chinese individuals aged >25 years had practically no knowledge of common periodontal prevention and treatment, with only a few undergoing more or less regular oral examinations (27). A subsequent study performed in 2012–2015 among 50,991 Chinese showed that 75% of them lacked periodontal knowledge and that 97% did not have regular scaling (28). The present study is the first to examine the KAP toward oral diseases among Chinese patients with oral diseases. The high KAP is probably because all participants had been diagnosed with oral diseases and underwent treatments for their condition, in contrast with the previous Chinese studies that were performed in the general population. Therefore, the participants in the present study are more likely to have received information about their disease and oral health in general and advice on proper oral care to prevent recurrence or the development of other diseases or sought information by themselves.

Supporting that view, the SEM analysis showed that the frequency of oral examination influenced knowledge and practice, suggesting that higher exposure to oral health professionals increased knowledge and improved practice. The participants are also likely to have sought information by themselves on the Internet or with relatives. In addition, most participants had a college/undergraduate education and a middle income. Socioeconomic factors are major determinants of KAP (29), and the present study might be biased due to the participants’ higher socioeconomic status than the general Chinese population. It is well known that socioeconomic status is a major determinant of health literacy (29). Nevertheless, the participants also represent the population of patients seeking oral care since patients with a lower socioeconomic status will often not undergo proper oral care (30, 31), as also observed in China (32, 33), Germany (34), Jordan (35), and Sweden (36). The SEM analysis also showed that expenses for oral disease per year influenced attitudes. Indeed, several studies showed that treatment costs were a barrier to proper oral health (37, 38). In the present study, two-thirds of the participants were ≤35 years old. Age is a major determinant of oral health (39), with older adults showing poor oral health. The young age of the participants in the present study could have contributed to the high KAP compared with previous studies.

A large study performed in the Chinese general population in 2012–2015 revealed that 2.6% were using floss at least once a day, 2.6% were undergoing scaling at least once a year, and 6.4% would visit a dentist in case of gingival bleeding (28). Since then, the Chinese government implemented policies to try to improve oral health in China (40), which could also have contributed to the high KAP observed here.

Nevertheless, this study has limitations. It was performed at a single hospital, leading to a small sample size and limited generalizability. The participants were enrolled through convenience sampling, which could introduce bias since only the interested individuals applied for participation. The online and offline participants were not compared. The questionnaire was designed by local investigators, and its content was probably biased by the local guidelines and policies, also limiting generalizability. In addition, the questionnaire did not undergo a formal validation process; it was not intended to be a standardized questionnaire but a survey. The study was cross-sectional, preventing the analysis of cause-to-effect relationships. Although the SEM analysis provides some information about the relationships among KAP dimensions and possible influencing factors, it is a statistical method that provides an approximation, at best. Finally, all KAP studies are at risk of the social desirability bias, which entails that some participants might answer that they do what they should do instead of what they are really doing (41, 42). Considering the high attitude and practice scores, that bias is probably active in the present study. In addition, KAP is subjective and reflects more on the intention rather than the actual execution.

In conclusion, Chinese patients with oral disease have sufficient knowledge, positive attitudes, and good practice toward oral diseases. Specific knowledge gaps and misconceptions were identified in the participants and would require improvements. Educational interventions should be designed to improve the KAP toward dental care further.

## Data availability statement

The original contributions presented in the study are included in the article/supplementary material, further inquiries can be directed to the corresponding author.

## Ethics statement

The studies involving humans were approved by the Ethics Committee of Tongji University Affiliated Stomatological Hospital ([2023]-SR-36). The studies were conducted in accordance with the local legislation and institutional requirements. The participants provided their written informed consent to participate in this study.

## Author contributions

WW: Conceptualization, Data curation, Formal analysis, Investigation, Methodology, Writing – original draft, Writing – review & editing.
